# Attributions of survival and methods of coping of long-term ovarian cancer survivors: a qualitative study

**DOI:** 10.1186/s12905-021-01476-1

**Published:** 2021-10-28

**Authors:** Dana Ketcher, Susan K. Lutgendorf, Susan Leighton, Marianne Matzo, Jeanne Carter, Arjun Peddireddy, Beth Y. Karlan, William P. Tew, Anil K. Sood, Eileen H. Shinn

**Affiliations:** 1 Department of Health Outcomes and Behavior, Moffitt Cancer Center, Tampa, FL USA; 2 Departments of Psychological and Brain Sciences and Obstetrics and Gynecology and the Holder Comprehensive Cancer Center, University of Iowa, Iowa City, IA USA; 3 Ovarian Cancer Research Alliance, Washington, DC USA; 4 Palliative Care Nursing, Survivorship and Supportive Care, Stephenson Cancer Center, University of OK, Oklahoma City, OK USA; 5 Gynecology Service, Department of Surgery, Gynecology Service and Department of Psychiatry, Memorial Sloan Kettering Cancer Center, New York, NY USA; 6 McGovern Medical School at The University of Texas Health Science Center at Houston (UT Health), Houston, TX USA; 7 Department of Obstetrics and Gynecology, University of California at Los Angeles, Los Angeles, CA USA; 8 Department of Medicine, Memorial Sloan Kettering Cancer Center, New York, NY USA; 9 Department of Gynecologic Oncology and Reproductive Medicine, The University of Texas, M.D. Anderson Cancer Center, Houston, TX USA; 10 Division of OVP, Cancer Prevention and Population Sciences, Department of Behavioral Science, The University of Texas, M.D. Anderson Cancer Center, Houston, TX USA; 11 Present Address: Memory Keepers Medical Discover Team, University of Minnesota Medical School, Duluth, MN USA

**Keywords:** Ovarian cancer, Long term survival, Well-being, Coping, Attributions, Meaning

## Abstract

**Background:**

Only 8–23% of advanced epithelial ovarian cancer patients survive for 10 years or longer. Given the need for targeted interventions to improve survival, we interviewed this relatively rare survivor population to gain personalized insights into the reasons for their survival. The aim of this study was to characterize subjective attributions of survival and specific coping mechanisms long-term survivors of ovarian cancer.

**Methods:**

Twenty-two semi-structured, qualitative interviews assessing survival attributions and coping strategies were conducted from April to November 2014. Data were analyzed in a multistep process using ATLAS.ti.8: codes were identified during review of the transcripts and refined with literature review; the frequency of codes and code co-occurrence was calculated, and codes were grouped into themes. Resulting themes were checked by a national leader of an ovarian cancer advocacy organization and compared against available literature.

**Results:**

Thematic analysis found that participants credited their long-term survival to a variety of factors including medical, social, religious/spiritual, and lifestyle/personal characteristics. Some participants rejected these same attributions, concluding that the reason for survival was due to luck or unknowable. Several of Carver et al.’s theoretical dimensions of coping were evident in our sample: planning, positive reinterpretation, social support, religion and acceptance whereas three relatively new strategies were uncovered: *conserving emotional energy, value-based activity coping*, and *self-care*.

**Conclusions:**

Long-term survivors’ perspectives were largely consistent with those of newly diagnosed ovarian cancer patients and ovarian cancer survivors of shorter duration. However, the long-term survivors were also willing to reject conventional attributions for survival and recognized the importance of disciplined self-preservational coping strategies.

**Supplementary Information:**

The online version contains supplementary material available at 10.1186/s12905-021-01476-1.

## Background

Ovarian cancer is relatively rare, affecting 1.3% of women in the U.S. [1, 2]. Owing to the lack of early detection strategies, approximately 80% of cases are diagnosed at an advanced stage when prognosis is markedly poorer [3, 4]. Recent SEER analyses estimate that only 23% of patients with stage III disease and 8% of patients with stage IV disease survive 10 years or longer [5–7]. Our group previously identified various clinical predictors of 10+ year survival including optimal cytoreduction and having platinum-sensitive disease [8]. Although molecular, clinical, and pathologic factors are important predictors of patient survival, it is becoming increasingly clear that biobehavioral and lifestyle factors may be important contributors to survival [9, 10]. However, little is known about long-term survivors’ subjective understanding of how and why they survived ovarian cancer, including explanations that go beyond the characterization of their treatment or disease. Whether survivors’ attributions are related to carcinogen exposure [11], genetics, medical treatments [12], or lifestyle factors, careful delineation of survivors’ subjective understanding can guide the development of future interventions to improve survival.

From the time of first diagnosis, most women with high-grade serous ovarian cancer face a chronic cycle of recurrence and/or progression, and remission, leading to physical and psychosocial stressors that may include chronic pain, neuropathies, fatigue, anxiety, and cognitive impairment [13]. Well past the initial treatment period, these ongoing challenges require continual and varied methods of coping [14, 15]. Therefore, having a long-standing interest in the influence of psychosocial stress on survival outcomes in epithelial ovarian cancer, we also assessed specific coping strategies for stressors related to ovarian cancer and other life stressors during the survivorship period. Folkman and Lazarus proposed two primary modes of coping with stressful situations: problem-focused coping, which is characterized by efforts to alter the original source of stress through behavioral response or by changing the environment related to the stressor, and emotion-focused coping, which regulates stressful emotions [16]. Individuals report using both coping styles when faced with difficult situations [17]. For problems that are controllable, problem-focused (sometimes called active coping) is considered helpful. For problems that are uncontrollable, such as poor prognosis, emotion-focused strategies such as acceptance, reinterpretation, and seeking emotional social support, may help mitigate the distress surrounding the stressor [17, 18]. In a review of 30 studies assessing patients in coping with incurable cancer, the most common coping strategies were emotion-focused (e.g. taking on a fighting spirit, positive reframing, spirituality, and acceptance) [15]. On the other hand, participants who had long-term cancer survival reported that planning, acceptance, and optimism were important coping strategies [11].

While qualitative work has been done with long-term cancer survivors of all types [19] and with ovarian cancer survivors, these studies have focused on issues other than long-term survival attributions, such as how cancer had affected their quality of life, [19] psychological well-being, [20] sexuality, [21] and perceptions of working relationships with healthcare providers [22, 23]. Other qualitative studies with ovarian cancer survivors were conducted earlier in the treatment trajectory at time of diagnosis [24]. One focus-group study, by Alimujiang and colleagues, did assess long-term ovarian cancer survivors’ attributions for exceptional survival of 5 years or more [25]. However, to our knowledge, qualitative analysis focusing specifically on survival attributions *and* coping with psychosocial stress has not yet been reported with long-term (> 10 years) ovarian cancer survivors. Therefore, we conducted open-ended, semi-structured interviews of patients with primarily high-grade, advanced-stage ovarian cancer who had survived 10 years or longer after initial diagnosis.

## Method

Twenty-one survivors with stage III-IV and one survivor with stage II serous ovarian cancer were recruited and enrolled onto the study between April and November 2014. Sample size was limited by time and resources; past research has found that between 6–12 participant interviews are sufficient to determine high-level themes and issues [26]. Patients were eligible for this study if they were at least 18 years of age, could speak and read English, were not on active treatment, were not in hospice, and had been diagnosed with high-grade epithelial ovarian, peritoneal or fallopian tube cancer at least 8 years prior to study enrollment. Patients were recruited either through online flyers on the national Ovarian Cancer Research Alliance list-serve or in-person during patient clinic visits at the following academic medical centers: Memorial Sloan Kettering Cancer Center and University of Oklahoma Health Sciences Center. All patients provided informed consent. Procedures were approved by the IRBs of all cooperating institutions.

### Procedure

For each participant, research staff scheduled 45-min semi-structured telephone interviews that were conducted by either a Ph.D.-level clinical psychologist (ES; JC) or a master’s-level counselor. Interview questions were initially drafted by a panel of psychosocial and gynecologic oncology experts, iteratively reviewed and resulted in nine questions about lifestyle, coping strategies, and attributions for long-term survival (see Additional file 1 for interview guide). Specific probes were used to find out more about the following domains: (a) relationships with a significant other(s); relationships with family members and friends; major stressful events relating to cancer, family, finances, and/or caretaking; (b) determining specific coping strategies for these events; (c) the impact of cancer on perceived life meaning and activities, and (d) attributions for survival. Each interview was conducted in one 30–60-min session, audio-recorded, and transcribed.

### Data analysis

Transcripts of participant interviews were analyzed in a multistep process using ATLAS.ti 8 Windows and following phases of thematic analysis described by Braun and Clarke [27, 28]: First, DK and AP independently familiarized themselves with the raw interview data. Next, a subset of the authors (ES, DK, AP, and SKL) jointly developed a thematic framework related to attributions of survival and methods of coping. DK and AP independently coded for attributions of survival and methods of coping and then brought coded transcripts to the other co-authors for review. Over the course of several meetings, these co-authors reviewed each coded transcript to capture all aspects of survival attributions and coping methods. Disagreements between researchers were used to refine codes and come to agreement about themes and further coding. As a further check for accuracy, all qualitative results were discussed with co-author SL, a long-term ovarian cancer survivor and national leader of the Ovarian Cancer Research Alliance advocacy organization [29].

Responses to the question, “What do you feel has been the most important factor contributing to your long-term survival?” were identified and coded for thematic analysis. Additionally, transcripts were coded for other participant responses that were not in direct response to the above question but were listed as a reason for survival.

To thematically organize responses for methods of coping with stress, the theoretical subscale structure of the COPE Inventory by CS Carver, MF Scheier and JK Weintraub [17] was used as a framework: emotion-focused coping was first differentiated from problem-focused coping and subdivided into the additional 11 dimensions suggested by Carver et al. (Table 1). Throughout our analysis, a general inductive approach was taken in order to identify additional coping strategies [30]. We also determined the frequency of codes and code co-occurrence to establish overarching themes.

**Table 1:** Example of codes and sub-codes for methods of coping and long-term survival

Code *Sub-code* Definition	Example
**Long term survival**	
*Medical* Of or relating to the practice of medicine, including host characteristics, treatment regimens, and disease characteristics	*“The quality of the care I received at [hospital]”* *“First is, I got my surgery done by a gynecologic oncologist. And second I was optimally debulked. Third is, I have the standard of care for time of taxol and carboplatin. And all of my chemo treatments were on schedule. None were delayed”*
*Lifestyle choices and personal characteristics* Activities and/or characteristics such as exercise and diet choices to choices about personality and attitude	*“I keep my body in good shape, so I work out and watch my diet. I eat organic”* *“I’ve always had a very positive attitude. I never really felt that anything could happen to me”*
*Social* Friendly companionship, connections, or relations	*“Support of my friends and family and members of my support group.”* *“Helping other people has helped me, make me feel like I can contribute and it’s been worthwhile*
*Religious/Spiritual* A focus on religious organization, ‘higher power’, or interior life of a person	*“God isn’t ready for me yet so he has let me live this long, I guess.”* *“Spirituality, you know…knowing that there’s someone greater than us”*
*Struggling/searching for answers/meaning* Attempting to understand one’s life and experiences	*“I wish I knew that answer”* *“I’ve kind of come to the conclusion that it’s rather random”* *“I believe that part of being a long-term survivor is luck”* *“When I got cancer I never asked why I got it, but I do ask why me? You know, why am I surviving 11 years?”*
**Coping** *Conserving emotional energy* A choice to disengage from stressful situations or people	*“I’m not willing to put up with toxic people or BS”* *“I’m real quick to remove people from my life who are not supportive”* *“When I got the diagnosis I said, I can only take so much information at a time and I’ll tell you when I’m ready to hear the stage, and I knew it was probably stage three or four but I didn’t think I could hear four, and so I said, ‘I’ll let you know when I’m ready’”*
*Value-based activity* Any activity which brings joy, reduces stress, or has some other positive impact on the individual	*“I started doing volunteer work at a nonprofit and then it became a job for me. Love it to pieces”* *“We have a sailboat…we spent each spring into June, we spent time in the Bahamas on our sailboat, living on it. If things just get a little too crazy, we are on a bay and we can take our sailboat out and it’s kind of like going to the psychiatrist…that relieves our stress”*
*Self-care* Any activity deliberately done to address mental, emotional, and physical health	*“I decided today that I needed to rest, let my body kind of rest, so I got up this morning and I showered and I got right back into bed”*
Planning* Involves creating strategies and steps in order to handle the stressor or problem	*“It’s just one day at a time. Today we go to this doctor, tomorrow I need to get groceries and make sure we have the health things on hand for him [husband]”*
Active coping* Involves taking steps to remove, bypass, or improve a stressor	*“I didn’t want to sit around and be a victim, boohoo me, so instead I decided to learn all I could about this disease”*
Positive reinterpretation and growth* This method of coping aims to approach the stress by interpreting a stressful situation in a more positive way	*“That’s been a key factor for me and an ability to give back and feel, perhaps, less guilty about surviving when others did not”*
Use of instrumental social support* Seeking advice, assistance, or information	*“Right after I finished the six rounds of Taxol and cisplatin, I went to see an art therapist…I was really able to deal with my fear of recurrence most effectively with that”*
Use of emotional social support* Receiving moral support, sympathy, or understanding	*“As soon as I was able to, I began going to this twice a month support group…I see them as my pack”*
Religious coping* Religion may function in different ways to enhance coping under stress by providing emotional support, positive reinterpretation and growth, or active coping	*“I think you relax a little bit and you, um, I don’t know, there are certain things you resign yourself to and I was getting better at giving things to God rather than fighting”*
Acceptance* An important method of coping in situations where the stressful event must be accommodated rather than easily changed	*“We’re all gonna die so let’s put that in perspective”* *“Worrying is not going to change it so, I’m not going to worry about it”*

*Coping code definitions adapted from Carver et al. [15]

## Results

### Participant characteristics

A total of 22 interviews were conducted, but demographics were only completed by 19 participants (Table 2). The sample was largely White (90%), Non-Hispanic (100%), and retired (52.6%), with most participants having completed college or a post-graduate education (68.5%). On average, women in this sample were diagnosed at 49.9 years old (range 36–68 years) with stage III ovarian cancer (84.2%) and were in remission (42.1%) at the time of study entry. Average age at the time of interview was 66.1 (± 7.5) years. All women in this study were at least 10-year cancer survivors (M = 15.1 (± 5.01 years).

**Table 2:** Participant demographic characteristics

	M or freq (range)	% or SD
Age at interview	66.1 (53–79)	7.479
Age at diagnosis	49.9 (36–68)	8.202
Survival time (years) (N = 22)	15.1 (10–27)	5.01
*Race*		
White	18	90.0
Native American	1	5.0
Other	1	5.0
*Recruitment location (N* = *22)*		
Memorial Sloan Kettering	4	18.2
Ovarian Cancer Research Alliance	15	68.2
University of Oklahoma Health Sciences Center	3	13.6
*Ethnicity*		
Non-hispanic	19	100
*Stage at diagnosis*		
Stage II	1	5.3
Stage III	16	84.2
Stage IV	2	10.5
*Cancer grade*		
High grade	6	31.6
Low grade	2	10.5
Not sure	11	57.9
*Histology*		
Serous	14	73.7
Non-serous	1	5.3
Not sure	4	21.1
*Current status of cancer*		
I have completed primary (first line) treatment AND my cancer is in remission. I am no longer on treatment	8	42.1
I have completed primary (first line) treatment AND my cancer is currently in remission. I am currently on a regular treatment to keep it in remission	1	5.3
I completed primary (first line) treatment but my cancer came back. I am currently on treatment for this	1	5.3
Other	9	40.9
*Education*		
Some college/technical school	6	31.6
Four-year college or university	4	21.1
Graduate school/professional school	9	47.4
*Employment status*		
Employed outside of the home	7	36.8
Homemaker	2	10.5
Retired	10	52.6
*Annual household income*		
Less than $25,000	1	5.3
$25,001—$50,000	6	31.6
$50,001—$75,000	3	15.8
$75,001—$100,00	5	26.3
Greater than $100,000	4	21.1
*Current marital status*		
Married	13	68.4
Living with partner	1	5.3
Divorced	4	21.1
Widowed	1	5.3

*N* = 19 unless otherwise indicated

### Attributions of survival

In response to the interview question, “What do you feel has been the most important factor contributing to your long-term survival?” answers were coded under five main themes: medical, lifestyle choices and personal characteristics, social support, spiritual/religious, and unknown (searching for meaning, search for answers). Some participants had multiple explanations for their longevity and these were all coded separately.

#### Medical

Nineteen participants cited aspects of medicine and medical care as perceived reasons for their longevity. Certain medicines (e.g., Celebrex), clinical trials, and specific treatment regimens (e.g., intraperitoneal chemotherapy) were also mentioned as perceived reasons for long-term survival. Additionally, optimal debulking, specific chemotherapy regimens and surgical skill were cited. One 13-year survivor described her surgeon thus: “He [my surgeon] was really aggressive and meticulous.” (S13).

#### Lifestyle choices and personal characteristics

Women described a variety of choices, including exercise, diet, supplements, and avoiding stress, as reasons for their long-term survival. Other participants spoke about combining humor and a positive attitude with either a determination to survive or spirituality, or both, as secondary reasons for long-term survival. For instance, one woman stated, “Well, three things were kind of major things. I was referred to a gynecologic oncological surgeon…who was a hot shot. And, I think probably my sunny nature and basic optimism,” (S17, 16-year survivor). Having the right genetics was also included as important to survival, with one woman grandparents and great-grandparents that lived into their 90 s. In all, 14 (63.6%) women mentioned lifestyle choices and/or personal characteristics as important for their survival.

#### Social support

Twelve participants (54.4%) mentioned both the receipt of support from medical staff, support groups, family and friends as well as the opportunity to give back to others was integral to their survival. Even after ten years, one survivor (S05) singled out the importance of encouragement from her medical team: “absolutely amazing…I’d walk in the door and they’d tell me ‘Happy Wednesday!’ Everybody was so upbeat and it really made a difference.” Another participant (S21, 11-year survivor) described the “positive, supportive attitude I had from the company I worked for and the people that worked in that company” as having a “huge impact” on her cancer journey and recovery.

In addition to family and friends who provided integral support, participants cited the importance of helping newly-diagnosed ovarian cancer patients and volunteering in ovarian cancer advocacy organizations as a factor in their longevity. For one participant, who indicated that she had a dedicated phone line in her home for newly diagnosed women to call her, this “ability to give back” was helpful:

I would say that, you know, that’s been a key factor for me and an ability to give back and to feel, perhaps, less guilty about surviving when others did not. At least I’m, you know, reaching out, trying to help other people. Maybe that’s the reason I’m here. (12-year survivor, S02)

Others remembered that when first diagnosed, how important it was to stay alive for their children and “be there for them.”

#### Spiritual/religious

Twelve (54.5%) participants alluded to God, a higher power, and/or their spirituality as a reason for their survival. When asked to elaborate, one participant stated, “I would assume He’s just not ready to take me. I mean, I really, I have done nothing different in my life as far as making changes.” (10-year survivor, S22) In some instances, women included other reasons for survival (e.g., medical, social, etc.) in addition to their belief in God or a higher power.

#### Struggling for answers, searching for meaning

While most participants were able to make attributions for their long-term survival, it is important to note that five of the 22 cancer survivors were open to the idea that their survival was random, especially after comparing their experiences with other ovarian cancer patients. For these women, the implication that long-term survival was somehow under one’s control was annoying and false. One participant reported that it was a “pet peeve” of hers when people would ask why she had lived so long. In response to people telling her that `God had blessed her,” she said angrily:

What about my friends and my support group who have already lost their life? Because they wanted to live just as bad as I did…I honestly wish I knew [the answer to your question] because I have friends that have it [ovarian cancer] in my group and I wish I could tell them the answer to that. (10-year survivor, S08)

Other participants specifically rejected the idea that optimism, positive attitude, or health behaviors were explanations for long-term survival:

You know, a lot of people will say to me, ‘Oh, it’s your attitude’ or ‘You live a healthy lifestyle,’ that kind of thing. But, you know, I was in with these ten women and they all had wonderful attitudes and I thought they led healthy lifestyles and all of them have died. I can’t really say that there’s anything in particular that I do or that I have done. (15-year survivor, S09)

### Coping

Participants reported using many of the specific coping strategies described by Carver et al., including problem-focused coping strategies, as well as developing more emotion-focused strategies, such as acceptance, positive reinterpretation and growth, religion/spirituality, and engaging in social relationships (to both give and receive support). Examples of the following strategies are presented below.

#### Emotion-focused coping

Several participants displayed striking examples of *positive reinterpretation and growth* when faced with the initial stress of being diagnosed with a fairly lethal cancer. For example, participant S05 stated, “I was lucky because my husband had just passed away a month before I was diagnosed, so the rest of my family and friends really had to step in,” illustrating her choice to focus on the strengthening of bonds with friends and family rather than the unfortunate timing of her cancer diagnosis so soon after the loss of her husband. Another participant expressed that her own cancer was not BRCA1/2-related and thus not likely to increase risk for her children.

*Social support* was also important for both instrumental and emotional aspects of coping with the stress of cancer treatment. After 15 years, one survivor appreciated the depth of family members’ commitment to providing instrumental support:

One sister moved in with us at the time and she just stayed right here and cared for me for six months. I mean, she did the cooking and the shopping and so, I mean, everybody just stressed, you know, “Take care of yourself, get yourself well and we’ll do everything else,” and so she was absolutely marvelous. And the other sister who lives aways away was here as much as she could be and very, very helpful. (15-year survivor, S09)

Women reported that not only family members provided instrumental assistance, but various members of their community stepped up to help provide meals, help clean their home, drive them to their chemotherapy appointments, and take care of children. For some women, this was not an easy transition to let people help. One woman stated that:

I remember the first time … and watched a lady vacuum my whole floor. And it just about killed me, you know. I should be up there doing that, you know. But I learned. You have to learn to let people help you. (23-year survivor, S19)

*Religion* was also mentioned frequently as a way to cope with stress. One participant (S04, 15-year survivor) felt that she was not going through her cancer diagnosis alone because “angels were surrounding me…I kept them busy.” Another woman described prayer like “talking to a friend,” highlighting that even ritual features of religion could provide material social support. Prayer from friends was also described as a comfort, with one woman recounting the prayers as “lifting her up on the day or treatment”.

For others, religion may have been a conduit for *acceptance*, in that they would be “letting go and letting God” (i.e., letting God take care of their situation). One woman further explained:

I think when you have that mindset that, um, it’s ok, and that you’ve got Him on your side, it’s ok, that just helps ease a lot of anxiety I think, and all of that so that was, to me, a really big help. There’s something very comforting about your faith and I wish more people knew that. (10-year survivor, S11)

Some participants noted that the “unpredictability” of their cancer diagnosis helped change the way they thought about life, causing them to live more consciously in the present. As S07 described it, her philosophy is to, “be here now. Be brave. Help your friends with whatever they need even if they’re going to die tomorrow.” One participant (S08) explained that having lived with ovarian cancer for ten years lowered her expectations for a stress-free life, saying, “All those little bumps in the road…are all normal. I don’t have this expectation that life is supposed to be some fairytale.” Other participants described acceptance as a process over the years, moving from a natural response of anger and stress at being diagnosed to a state of “simplicity of accepting what I can’t change” et al.-Anon meetings.

#### Problem-focused coping

For some participants, problem-focused coping included *planning*. Participants spoke about gathering more information about their situation through ovarian cancer conferences and having risk-reducing surgeries completed (e.g., mastectomy). For others, planning was more introspective (e.g., writing in their journal) and/or mundane (e.g., going to the doctor, getting tests done), or enacted through sheer determination (“I’ve got to get up, I’ve got to do this, we’ve got to carry on”). *Active coping* (e.g., taking steps to eliminate a problem) was another coping strategy chosen by some participants as they redirected their energy into other projects related to their cancer diagnosis.

#### Unique coping strategies

While several of the coping strategies were aligned with Carver’s theoretical dimensions, our sample also described additional types of coping not previously well-characterized by other researchers. These strategies include *conserving emotional energy*, *engaging in value-based activities,* and engaging in *self-care*. Taken together, this additional constellation of coping strategies can be characterized as being a hard-won recognition of the importance of disciplined self-preservation.

C*onserving emotional energy* Knowing when to step away from situations that cause undue stress or sadness was cited by several participants as an important coping strategy for non-cancer related stress. For example, one 10-year survivor (S08) recounted that she would “no longer tolerate a brother that doesn’t tolerate me.” Another 14-year survivor (S07) stated that she was “not willing to put up with toxic people or BS”. Women stated that by removing whatever or whomever was causing “drama” in their life (i.e., stress), their quality of life was greatly improved. One survivor reported that removing stress from her life was a conscious decision on her part, and further elaborated why that was important to her:

I have a couple of friends…the moment they stop appreciating me as an individual and they try to change me or belittle me in any kind of way, I pull away immediately. I just know what it feels like and it’s like, you know…I’ll give them space and then I’ll come back and say, ‘This is why I pulled away’. We can regroup and move forward. … but I immediately pull away and... it’s just a self-preservation for myself. I just...I know my body cannot do that. I mean, just to cry over sadness makes my bones hurt, you know, and so I just...that’s one area that I’ll protect ‘till the cows come home...because to me that’s what kills you, is dealing with other people’s shit. (10-year survivor, S08)

The active decision to regulate information, people, and situations was viewed as a necessary and healthy coping mechanism by women in our study. Some managed cancer-related stress by controlling the timing and amount of information at the time of cancer diagnosis.:

When I got the diagnosis I said, ‘You know, I can only take so much information at a time and I’ll tell you when I’m ready to hear the stage,’ and I knew it was probably stage three or four but I didn’t think I could hear four and so I said, ‘I’ll let you know when I’m ready’ and, you know, it was maybe three or four weeks after my diagnosis that I was ready to hear that, but there was that period of time that I just didn’t want to hear that yet. (15-year survivor, S09)

Engaging in activities that gave their life joy and a sense of purpose was cited by several as a key coping mechanism. Many survivors pointed out the importance of finding activities that allowed for refocusing away from the natural inclination to persistently worry after being diagnosed: “It’s very hard for [women with ovarian cancer] not to think about it every minute because they can go to the computer [and read about ovarian cancer on the internet].(**S06)”** Participants made the distinction of activities that kept them busy (which could be construed as an avoidant coping response), versus staying busy with activities that provided meaning. Several women pointed out that *value-based activities* included participating in their children’s school activities, hiking, art, writing, traveling, starting support groups for newly-diagnosed women, and volunteering with nonprofits. One woman described the importance of her advocacy activities:

I didn’t want to sit around and be a victim, boohoo me, so instead I decided to learn all I could about this disease. Advocate for myself and advocate for others. So, um, I’d go to Capitol Hill. I’m an advocate leader for [ovarian cancer advocacy group]. And next month I’m going to be training a group of women from [State] and we’ll go visit our legislatures. And, you know, too many other women are dead, or too sick, or too broke from treatments to go to Capitol Hill, so, even though I’ve had recurrences, I’m still really lucky I’ve had a pretty darn good quality of life. So, I have to make my voice count for those who can’t be there. (14-year survivor, S07)

Additionally, the activities that women described to us were sometimes forms of *self-care*, enacted in order to bring joy and relieve stress. These activities were important for addressing mental, emotional, and physical health. For one 23-year survivor (S10), she listened to her body’s needs and one day “decided that I need to rest, let my body kind of rest, so I got up this morning and I showered and I got right back into bed.” For others, physical activity (such as walking) provided a sense of calm and some relief from anxiety. Still others cited yoga, acupuncture, and meditation to deal with problems and stress in their life. Another 14-year participant described having learned to not care whether these activities were seen as selfish, saying, “There’s nothing like a brush with death. You know, it’s a real wakeup call about how is it that you want to live your life. So, I think I’m less concerned about what other people think and just willing to follow my own path.” (**S07**).

## Discussion

Due to the rarity of both ovarian cancer incidence as well as the low odds of surviving 10 years with high-grade epithelial ovarian cancer, there is little information about the hard-won perspectives of this specific sample. Several of our findings are consistent with the careful work done by Alimujiang et al., whose focus group members identified strong social support, lifestyle factors, and having a strong sense of life purpose as important reasons for their survival [25]. Based perhaps on differences in interviewing technique (i.e., one-on-one) and sample treatment characteristics (all of our participants had at least 10 years survival) our study did uncover some unique findings. When asked why they believed they had survived for 10 years or longer, a significant minority of participants described guilt over having survived, anger at family and friends for proclaiming that positive attitudes and/or health behaviors explained their exceptional survival and were willing to concede that there was no answer. Religious participants who did attribute their survival to a specific reason (God), displayed a similar type of unwillingness to take credit for their exceptional survival. These findings can inform the tone of future biobehavioral interventions targeting increased survival for ovarian cancer patients by emphasizing the limit to current medical knowledge and being careful to avoid over-promising as to any intervention’s potential benefit.

Unlike Alimujiang et al.’s finding that the focus groups displayed a strong sense of positivity, a positive attitude was not uniformly endorsed by all of the participants in our sample, especially in the group that was still “searching for meaning.” It has been noted by others that the pressures for patients to exhibit positive thinking and attitudes can have unintended consequences, such as a sense of failure or guilt if the patient does not get better [31] or marginalization of negative feelings, which comes with its own psychological consequences [32]. Endorsement of negative feelings among our participants is a reminder that it is important to distinguish between survivors’ desire for authentically positive and meaningful experiences versus well-intentioned but unreasonable expectations.

In addition to the dimensions of coping suggested by Carver and colleagues, [17] our qualitative analysis uncovered additional forms of coping, which we describe as *conservation of emotional energy*, *engagement in value-based activities,* and *self-care.* We believe that these disciplined forms of self-values were developed over time, and in response to facing mortality in themselves and their fellow ovarian cancer patients. Rather than viewing these strategies as *strictly* avoidant, it is apparent that survivors are actively regulating stressful situations or prioritizing self-affirming activities. While avoidant coping has been found to be adaptive in the short-term but counterproductive long-term [33], the women in this study expressed the belief that the conscientious regulation of their emotional energy was essential to supporting their well-being in the face of coping with ovarian cancer. Thus, while these strategies may initially look like avoidance, it is apparent that women are exerting their individual agency in precise ways to confront, control, and mitigate stressful situations wherever possible. For example, as a way to combat persistent worry and self-rumination, engagement in value-based activities and self-care activities emerged as an important activity to support physical, mental, and social needs [34]. Similar to findings from Kidd and colleagues [35], it is apparent that self-care activities are also important in maintaining a sense of normalcy in women’s day-to-day lives. Self-care and other value-based activities should be considered important in long-term cancer survivorship, and indeed have long been recommended for survivorship care plans [36].

Several studies have found that when individuals are faced with a traumatic event such as cancer, they must adapt their worldview to make sense of the change and work to integrate that event. This process, known as meaning-making, provides a way to understand how individuals make sense of change, how they adapt their worldview to a traumatic event (such as cancer) and/or integrate that event [37]. For instance, in their study of cancer survivors, Park et al. found that meaning-making is a dynamic process which fluctuates over time [38]. For our participants, meaning-making was evident in several areas. For instance, participants in our study found enhanced meaning through relationships and experiences similar to results from van der Spek and colleagues [39] and Alimujiang et al. [40]. Several participants cited the importance of engaging in meaningful activities that promoted more positive outlooks. That meaning-making is so strongly present among our sample may indicate, just as it did in Park et al.’s study and others [37, 39, 41, 42], that meaning-making may be essential for healthy, long-term adjustment and coping with cancer survivorship.

*Limitations*. Our sample was small, with only 22 participants, and primarily white non-Hispanic, married, well-educated, with above average annual household income. Thus the findings in our study may be spurious and not generalizable to long-term ovarian cancer survivors who do not share the same characteristics as our sample. It is important that future work include a more diverse sample in terms of race, background and treatment setting. Another limitation is that since the participants who were recruited through the advocacy network, we did not have ready access to detailed disease and treatment information beyond having stage II-IV disease at diagnosis. Lastly, we did not assess *BRCA* mutation status, which could be an important component of women’s survivorship and coping.

## Implications and conclusion

Overall, it is apparent that ovarian cancer survivors in this study placed a great deal of importance on emotion-focused coping as a way to manage both the proximal and distal outcomes of cancer, in ways that are context- and person- specific. While healthcare professionals may regard emotion-focused coping strategies as outside the treatment domain, understanding the value that patients place on managing stress, purposeful activities, social support and positive reframing sets the stage for providers to encourage activities and organizations that support these values. Qualitative analysis shows that long-term ovarian cancer survivors have a unique psychological profile that may be characterized by a healthy skepticism of conventional attributions of survival such as health behaviors, a clear-eyed pragmatism and willingness to reject cultural norms of tolerating emotionally draining people within their network. While some reviews indicate that survivorship care plans may have few measurable benefits, [43, 44] our results may provide avenues of further exploration as to how well existing care plans address the hard-won insights provided by long-term ovarian cancer survivors. Future interventions should fully incorporate the unique perspective of long-term survivors; doing so will inform the timing of intervention strategies, provide a context for appropriate versus ineffective messaging, and be built upon the values of ovarian cancer patients/survivors.

## Supplementary Information

**Additional file 1:** Long-term Survivors Interview Form.

## Data Availability

The datasets used and/or analyzed during the current study are available from the corresponding author on reasonable request.
