# Supplementary material for: Attributions of survival and methods of coping of long-term ovarian cancer survivors: a qualitative study
Source: BMC Womens Health. 2021 Oct 28;21:376. doi: 10.1186/s12905-021-01476-1 (PMC8554947; doi:10.1186/s12905-021-01476-1)
Supplement: Supplementary file 1 — Additional file 1: Long-term Survivors Interview Form. [file 12905_2021_1476_MOESM1_ESM.docx]

**Long-Term Survivors Interview**

We would like to ask you some questions that will help us understand how people’s approach to life and lifestyle factors may contribute to their long term survival with ovarian cancer.

1. What do you feel has been the most important factor contributing to your long term survival? What are other factors that you also consider important? good docs, aggressive tx, low stress, good attitude, on clinical trials, not working, Good support system, healthy prior to cancer, cancer not in nodes, family history of longevity, support groups, working with OCNA, supporting others, quit toxic job, faith-based life, family supt, will to live, luck, willing to do anything to be cured, exercise, random, “IJ wish I knew”, changed my life to be drama-free, diet/supplements (listed), lots of chemo, “not really”, young and healthy, luck, blessed, God isn’t through with me, cancer caught in time, caught early, “living and loving to the fullest extent possible”
2. How long have you been/were you married or living with a partner?
   1. Has your marital/relationship status changed prior to or since your cancer diagnosis?
   2. If you were in a relationship when you were diagnosed, did it change after diagnosis?
   3. How did it change?
   4. Did you experience sexual changes?
   5. Did your relationships with other family members change?
   6. How so?
3. Have your relationships with friends changed since your diagnosis? How so?
   1. Some people feel that their friends abandoned them after diagnosis. Has that been true of you?
   2. Do your close friends share a diagnosis of ovarian cancer?
   3. Who do you turn to for support and what kind of support is most important to you?
4. Have there been any major stressful events that have occurred since your diagnosis? Any chronic stressors?
5. (Probe): Cancer-related stressors?
6. Family health-related stressors?
7. Family conflicts?
8. Financial issues?
9. Caretaking for another person for illness or other reason
10. For each stressor: How stressful was it? How long did the problem last?
11. For the stressful events discussed above: How long did it take you to “bounce back” emotionally? What strategies did you use?

- Probe: How did you use (specific strategy) to solve (problem)? How did you know it was working?

1. Did you experience any major stressors or trauma before diagnosis?
2. What are the important things that give your life meaning? Did cancer change the way you think about your life? If so, how was it before, and how do you think about your life now? Did cancer make it easier/harder to find meaning in life?
3. How important is your spirituality or connection with a religious faith to you?

Probe: Did your spirituality or connection to your religious faith or belief change after you were diagnosed? If yes, how?

After the two-year mark?

After the five-year mark?

=

1. What sports or exercise did you do before your diagnosis? If you did sports or exercise, how many minutes did you do this each week? Since your diagnosis, have you made any changes in your exercise? If so, please describe the changes. What kind of changes did you make? When did you make these changes?
2. How about changes in what you eat or the way you eat – has that changed since your diagnosis? If so, please describe the changes you made, and when you made the changes?
3. Is there anything we forgot to ask about that you think has helped you survive?
4. Are you able to retrieve your medical records?
